# Prion Protein Polymorphisms Affect Chronic Wasting Disease Progression

**DOI:** 10.1371/journal.pone.0017450

**Published:** 2011-03-18

**Authors:** Chad J. Johnson, Allen Herbst, Camilo Duque-Velasquez, Joshua P. Vanderloo, Phil Bochsler, Rick Chappell, Debbie McKenzie

**Affiliations:** 1 Department of Comparative Bioscience, University of Wisconsin, Madison, Wisconsin, United States of America; 2 Centre for Prions and Protein Folding Diseases, University of Alberta, Edmonton, Alberta, Canada; 3 Department of Agriculture Food and Nutritional Sciences, University of Alberta, Edmonton, Alberta, Canada; 4 Wisconsin Veterinary Diagnostic Laboratory, University of Wisconsin, Madison, Wisconsin, United States of America; 5 Department of Biostatistics and Medical Bioinformatics, University of Wisconsin, Madison, Wisconsin, United States of America; 6 Department of Biological Sciences, University of Alberta, Edmonton, Alberta, Canada; Creighton University, United States of America

## Abstract

Analysis of the *PRNP* gene in cervids naturally infected with chronic wasting disease (CWD) suggested that *PRNP* polymorphisms affect the susceptibility of deer to infection. To test this effect, we orally inoculated 12 white-tailed deer with CWD agent. Three different *PRNP* alleles, wild-type (wt; glutamine at amino acid 95 and glycine at 96), Q95H (glutamine to histidine at amino acid position 95) and G96S (glycine to serine at position 96) were represented in the study cohort with 5 wt/wt, 3 wt/G96S, and 1 each wt/Q95H and Q95H/G96S. Two animals were lost to follow-up due to intercurrent disease. The inoculum was prepared from Wisconsin hunter-harvested homozygous wt/wt animals. All infected deer presented with clinical signs of CWD; the orally infected wt/wt had an average survival period of 693 days post inoculation (dpi) and G96S/wt deer had an average survival period of 956 dpi. The Q95H/wt and Q95H/G96S deer succumbed to CWD at 1,508 and 1,596 dpi respectively. These data show that polymorphisms in the *PRNP* gene affect CWD incubation period. Deer heterozygous for the *PRNP* alleles had extended incubation periods with the Q95H allele having the greatest effect.

## Introduction

Chronic wasting disease (CWD) is a prion disease affecting *Cervidae*. Currently, CWD is pandemic in both domestic and wild deer. Over the last decade, the geographic distribution of CWD has increased, spreading from Colorado and Wyoming across North America. In areas where CWD has become endemic, disease incidence continues to rise. CWD agent persists in an infectious form in the environment. Cohabitation of contaminated regions with disease-naïve *Cervidae*, as well as susceptible species such as ovines, bovines and wild rodents, could extend the impact of CWD. Additionally, since cervids are the predominant game and subsistence species for meat, the risk of CWD transmission to humans cannot be ruled out. Unlike Bovine Spongiform Encephalopathy (Mad Cow disease), CWD is contagious and can spread horizontally through contaminated environments. As a result, the susceptibility of the host species is of great concern to the expansion of the epidemic as well as to the accumulation of infectivity in the environment.

Like other prion diseases, CWD agent replication involves the conversion of normal cellular prion protein (PrP^C^) to a protease-resistant disease form (PrP^CWD^). In sheep, mouse and human TSEs, it has been well established that amino acid polymorphisms in the prion protein influence susceptibility to disease agent [1], [2], [3]. Genetic analyses of various cervid populations in which CWD is endemic have also suggested that prion protein polymorphisms affect susceptibility [4], [5], [6]. Our study of hunter-harvested, CWD-positive white-tailed deer in the CWD-endemic area of southern Wisconsin demonstrated a significant difference in the incidence and histological presentation of CWD in deer with at least one Q95H or G96S allele [4]. Without, however, knowing the time, route or level of exposure of these free-ranging, hunter-harvested deer to CWD agent, the precise impact of these alleles on disease progression and susceptibility could not be assessed.

To test the hypothesis that *PRNP* genotype affects CWD disease progression and susceptibility, we initiated an experimental oral infection of white-tailed deer. Deer homozygous for the wild-type prion protein succumbed to CWD infection significantly earlier than deer heterozygous for or lacking the wild-type prion allele.

## Results

To determine the effect of *PRNP* polymorphisms on susceptibility to, and incubation time of CWD, twelve white-tailed deer, of known *PRNP* genotypes, were orally dosed with a defined CWD inoculum from hunter harvested deer. All deer were obtained as fawns from northern Wisconsin, a region with no cases of CWD as determined by extensive statewide sampling performed by the Wisconsin Department of Natural Resources (http://prodoasext.dnr.wi.gov/inter1/pk_cwd_zonerpt$.startup).

The deer in this study had *PRNP* alleles that were variable at amino acids (AA) 95 and 96 with the most common allele (17/24) having glutamine at position 95 and glycine at position 96, referred to as wild-type (wt). Two other alleles were present, a glutamine to histidine change at position 95 (Q95H) and a glycine to serine at position 96 (G96S). Six of the animals were homozygous for wt/wt *PRNP* alleles; 4 were wt/G96S heterozygotes, one was a wt/Q95H heterozygote and one was heterozygous for both the 95 and 96 polymorphisms (Table 1). Silent amino acid changes and the presence/absence of the *PRNP* pseudogene are noted in Table 1. Silent single nucleotide polymorphisms and/or the presence of the pseudogene were not linked to changes in rate of disease progression.

**Table 1:** *PRNP* locus traits of orally challenged white-tailed deer.

Fawn	NA 315/AA 95	NA 316/AA 96	Pseudo- gene	NA 90	NA 183	NA 354	NA 468	NA 585	NA 802
1							HOM	HET	
2							HOM	HOM	
3			yes				HOM		HET
4		HET	yes				HET	HET	
5							HOM		
6	HET			HET		HET	HOM		
7		HET					HOM	HET	
8							HOM		HET
9			yes		HET		HOM		
10		HET					HOM	HET	
11	HET	HET		HET			HOM	HET	
12		HET					HOM	HOM	

Silent polymorphisms, the presence or absence of the pseudo-gene and amino acid substitutions linked to disease progression (highlighted) are listed for each animal. Only one animal presented both amino acid substitutions in different alleles at the same time (Bold font).

### Incubation periods

**Figure 1:**
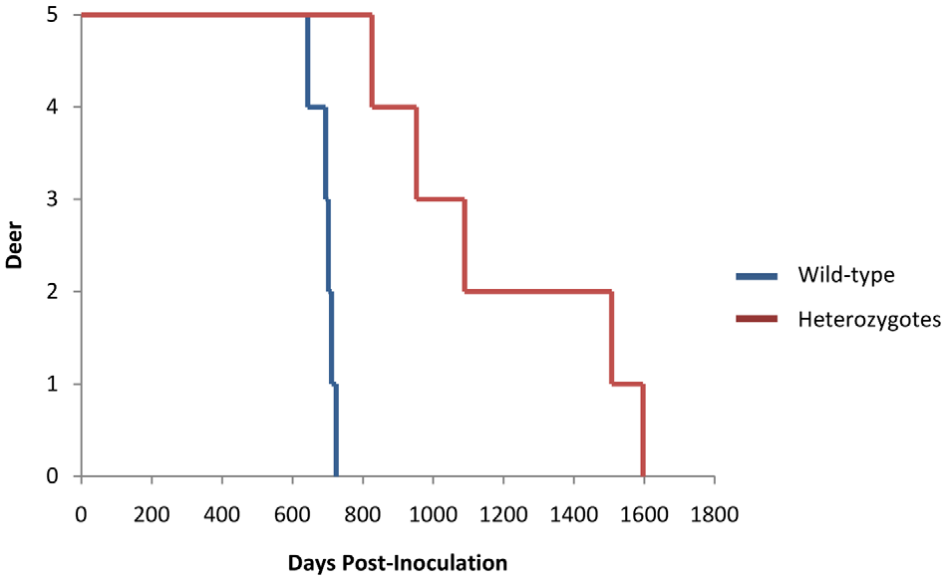
Survival curve of white-tailed deer orally challenged with CWD agents. Animals with only wild type alleles (blue) succumb to disease faster than individuals carrying at least one of the polymorphisms in positions 95 and 96 (Red). Wt/Q95H and Q95H/G96S animals present longer incubation periods and succumb to disease at similar times.

The major objective of this study was to determine the effect of prion protein polymorphisms on the rate of disease progression. Two deer were lost to intercurrent disease prior to onset of overt clinical signs of CWD. One animal, a wt/G96S amputee, was euthanized at 79 dpi due to non-CWD related physical problems. The second deer, a wt/wt animal, had an acute clostridium outbreak in the gut and was lost from the study at 416 dpi. The remaining 10 deer all presented with clinical signs of CWD (described in the following paragraph). The other wt/wt deer all presented with clinical CWD with an average incubation time of 693+/−27 days. All three G96S/wt deer succumbed to disease with an average incubation time of 956+/−107 days. Deer with one copy of the Q95H allele survived for a much greater period with Q95H/wt deer reaching end-stage CWD at 1,508 dpi and Q95H/G96S at 1,596 dpi (Figure 1).

Group differences were highly significant at the overall p = 0.00001 level. Each of the three pairwise group differences was significant at the 0.005 level or smaller. Thus we conclude that homozygous wt alleles are associated with shorter life expectancy and that Q95H appears to be associated with greater survival than Q96S. This second conclusion is a weak one because it was based on a comparison of only two Q95H/other (one G96S, one wt) deer with three G96S/wt alleles. No further statistical inference could be established regarding the effect of the individual Q95H or G96S allele due to small sample size.

### Clinical signs of CWD positive deer

**Figure 2:**
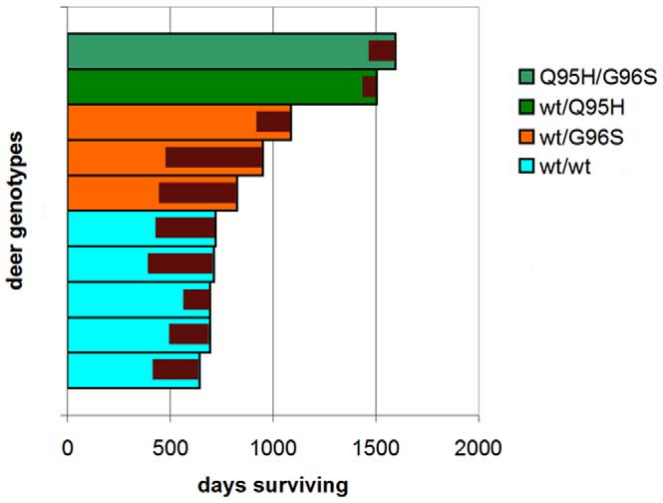
Incubation period and duration of clinical period. Internal red bars indicate clinical disease. Day zero is the day the oral inoculations were initiated.

The progression of CWD clinical signs in the orally infected white-tailed deer was variable and, initially, very subtle in individual deer. Signs were similar to those described for captive mule deer [7]. Early signs included brief loss of awareness, diet and behavioral changes, pronounced arching of the back, increased hyperexcitability and weakness or ataxia (more pronounced in the hind limbs). These signs were often subtle and transient, preceding overt clinical signs by four to thirteen months (Figure 2). Arching of the back was the most pronounced and common early symptom occurring up to nine months prior to advanced clinical signs. The progression from early clinical signs was generally characterized by a period of weight loss and reduced food consumption, increased ataxia and reduction in awareness. More advanced disease was marked by periods of odontoprisis, polydipsia and difficulty swallowing. An increase in fresh fruit and vegetables would generally stimulate increased food consumption and stabilize the individual deer's weight for two to three weeks. Progression through disease signs was occasionally abrupt. One deer (wt/wt) had very little weight loss and only three days of depressed appetite prior to recumbence (at which time it was euthanized). This may reflect the myriad of mechanisms by which CWD progression leads to death, a process more variable in cervids than inbred rodent models.

Overt clinical signs included pronounced ataxia, head tremors, advanced weight loss, increased difficulty swallowing, excess salivation, decreased coordination, decreased awareness, lethargy and regurgitation. One deer also displayed polyuria. Five deer displayed polydipsia with increased drinking but decreased efficiency of water intake. Once overt clinical signs were established and persisted for a week, the animal was euthanized. Although there were no obvious differences in the overt clinical presentations between deer with different *PRNP* genotypes, the duration of the clinical phase was much shorter in the heterozygous animals (Figure 2).

### PrP Immunohistochemistry

**Figure 3:**
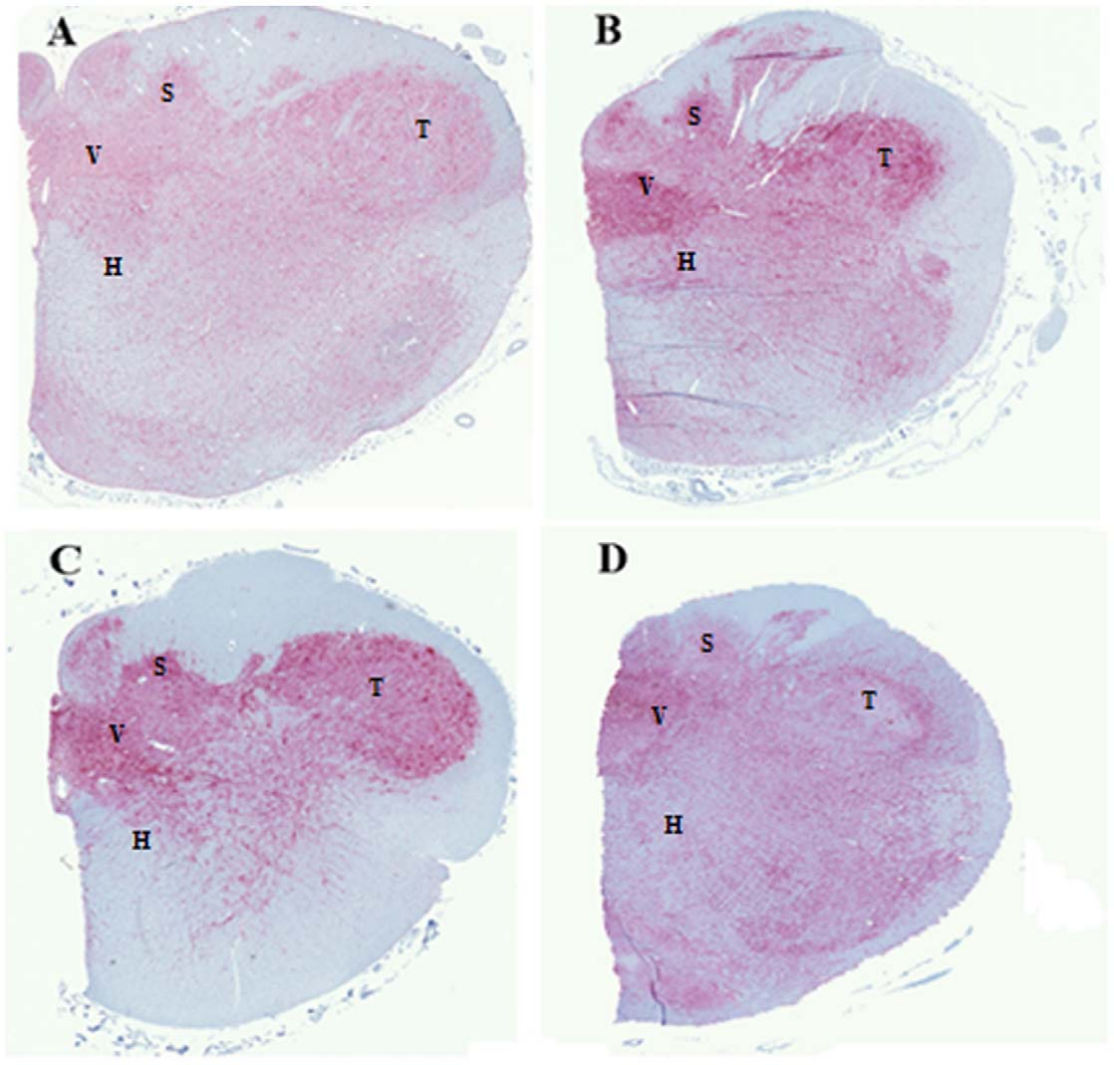
Immunohistochemical detection of PrP^CWD^ in the obex region of white tailed deer infected with CWD: A) wt/wt, B) wt/G96S, C) wt/Q95H and D) Q95H/G96S. Regions are indicated as: V = dorsal nucleus of the vagal nerve; S = nucleus of the solitary tract; T = spinal trigeminal nucleus and H = hypoglossal nucleus.

Brain, retro-pharyngeal lymph nodes (RPLN), tonsil, spleen and the ileocecal junction, were collected from deer following euthanization. Immunohistochemical staining of the medulla at the obex detected high levels of PrP^CWD^ staining in all clinically affected genotypes (Figure 3). The PrP^CWD^ staining pattern differ between each genotype, wt/wt animals presented diffuse bright-red chromogen distributed all over the tissue while Q95H/G96S, wt/G96S and wt/Q95H animals presented abundant staining comprising the nucleus of the solitary tract, the dorsal nucleus of the vagus nerve, the spinal trigeminal nucleus and the hypoglossal nucleus. Tissues from the deer lost to intercurrent disease were also analyzed. The wt/G96S heterozygous animal sacrificed at79 dpi was weakly positive for PrP^TSE^ in the tonsil, Peyer's patches and RPLN. No PrP staining was observed in the spleen or obex region of this deer. The wt/wt deer lost at 416 dpi had extensive lymph node staining for PrP and was stage three positive in the obex region of the medulla (data not shown).

### PrP^CWD^ immunoblot analysis

**Figure 4:**
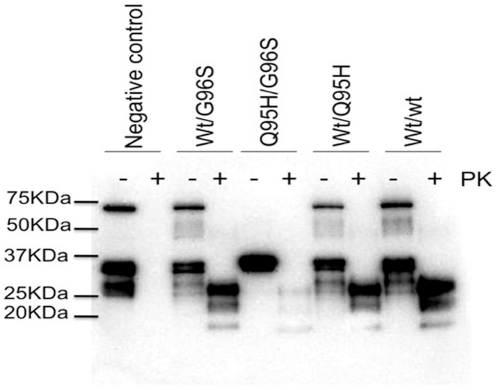
PrP^CWD^ electrophoretic patterns in different *PRNP* backgrounds. Brain homogenates with (+) or without (−) PK digestion were resolved by western blot using monoclonal antibody 8G8.

Brain homogenates prepared from the brains of clinically affected deer of each genotype were treated with proteinase K (PK) and analyzed by western blot (Figure 4). PK- resistant material was observed in all infected animals. Animals with at least one wt allele displayed similar banding patterns regarding intensity, glycoform ratio and molecular weight of the PK resistant fragments. Unlike the other genotypes, the Q95H/G96S animal had a lower molecular weight unglycosylated fragment; additionally, the intensity of the PK-resistant fragments was weak suggesting that the PrP^CWD^ produced in this genetic background is more sensitive to PK digestion. Interestingly, the glycoform ratio of the nonPK-digested sample from the Q95H/G96S animal differed from the other genotypes, lacking the mono- and un-glycosylated PrP isoforms. After protease digestion, however, the signal for these glycoforms is clearly present.

## Discussion

This study has directly addressed the impact of three white-tailed deer *PRNP* alleles (wt, Q95H and G96S) on the rate of progression of CWD to clinical disease stage in white-tailed deer. Deer homozygous for the wt/wt alleles present with and succumb to CWD more rapidly than heterozygous deer, consistent with the association of certain alleles with increased disease incidence in free-ranging white-tailed deer in Wisconsin [4]. This experimental infection demonstrates that the observed bias in *PRNP* allele frequencies in CWD-positive free-ranging deer is based on prion protein primary sequence.

The extended incubation periods observed in wt/G96S heterozygous animals and those animals with a Q95H allele suggest a reduced susceptibility of these alleles to conversion. The Q95H allele also provided a higher level of resistance to infection than the G96S allele. The deer heterozygous for the Q95H allele did not succumb to CWD until 1,508 and 1,596 dpi, with the first being euthanized more than 2 years after the last wt/wt deer was overtly clinically positive and 419 days after the last G96S/wt deer was euthanized. The presence of the Q95H allele is associated with a doubling of symptom-free incubation period via the oral route of infection since the onset of clinical signs for the wt/wt and G96/wt deer were 482 and 630 dpi, respectively, and early clinical signs were not noted in the Q95H heterozygous deer until 1,465 dpi. Most interestingly, the Q95H/G96S heterozygote deer did not substantially outlive the Q95H/wt animal, suggesting that genetic resistance is not additive [4].

Incubation times for wt/wt deer in this study were similar to those published for oral route in mule deer in a previous study [8]. The longer incubation periods associated with the presence of the Q95H allele or the G96S allele were not due to conversion incompatibility because the Q95H/G96S heterozygote succumbed to disease and the brain contained PK-resistant PrP^CWD^. The non-wild-type alleles could be linked to reduced PrP^C^ expression, as has been previously suggested [9], [10], however, this is unlikely given the minimal increase in incubation period observed with the Q95H/G96S heterozygote compared to the wt/Q95H animal.

Naturally infected, free-ranging deer less than 2 years of age have been identified as CWD-positive [11]. Given the dose of infectious agent provided to the deer in this study, it was anticipated that the incubation period would be similar, if not shorter than, that observed in the wild (∼2years). It is likely that the longer incubation periods we observed in the experimentally infected deer were primarily due to better care in captivity. The animals in this study were provided with food and health care; it is likely that the presence of clinical signs enhances extrinsic mortality in the wild. Clinical signs were noted as early as 436 dpi in our study and exposure to temperature changes, disease and nutrient availability may increase mortality due to secondary causes, predation, accident, and exposure.

There were differences in the duration and presentation of specific clinical signs in individual deer. *PRNP* genotype was not necessarily a predictor of clinical course. In the wt/wt and wt/G96S deer, with one exception, onset of clinical signs was similar. Differences were likely due to the individual characteristics of each deer. Deer with the shortest period of clinical signs tended to have a pronounced fight or flight response/anxiety, which may have masked early clinical signs. In Q95H, deer clinical course was relatively short with few early signs.

Although the presence of Q95H and G96S alleles resulted in extended incubation times, the impact of these alleles on disease transmission in free-ranging animals has yet to be determined. CWD agent has been identified in saliva [12], urine [13], and feces [14]. It is not, however, clear when infected animals begin shedding agent. It is possible that the longer incubation periods associated with the Q95H and G96S alleles will result in a more prolonged shedding of agent into the environment.

Electrophoretic profiling of the PrP^CWD^ showed similar glycoform ratio and molecular weights in all animals with at least one wt allele. The PrP^CWD^ from the Q95H/G96S animal, however, differed from the other genetic backgrounds with respect to molecular weight, level of PK-resistance and glycoform pattern suggesting that CWD agent produced in this animal may have different biological, biochemical and structural properties. The PrP^CWD^ generated by this animal would also appear to be different from the PrP^CWD^ present in CWD strains 1 and 2. These strains were identified by Angers et al [15] upon transmission of various sources of CWD agent from elk, white-tailed deer and mule deer into transgenic mice expressing the wt cervid gene. The PrP^CWD^ from strain 1 and strain 2 both had similar electrophoretic and glycoform profiles [15], resembling the wt/wt PrP^CWD^ in this study.

This study was conducted using CWD agent derived from hunter-harvested animals homozygous for the wt *PRNP* allele. Each animal received a total dose of 10 g of infected brain over 5 days. In natural infections, exposure to CWD agent would likely be sporadic and at a substantially lower dose. Thus, in natural infections, we would expect that disease incidence would be much lower with the two *PRNP* polymorphisms. The observation of a decreased incidence of CWD in deer with Q95H or Q96S *PRNP* alleles [4], [16] strongly suggests that decreased disease penetrance is the critical component of resistance of Q95H or Q96S deer to the naturally occurring infection. The minimal dose at which wt agent can penetrate heterozygous hosts is likely to be below the steady state level of agent in the environment.

In summary, polymorphisms in the primary sequence of the prion protein affect the incubation period of CWD infection in white-tailed deer. Animals homozygous for the wt allele have a greater rate of progression to clinical CWD disease than heterozygous animals. Transmission properties, incubation periods and clinical signs could be markedly different for CWD agent derived from these polymorphic PrP^CWD^ proteins.

## Methods

All fawns were tested for CWD by tonsil biopsy; all tested negative. The deer were housed individually in concrete rooms that had not been previously used for TSE studies. This study was carried out in strict accordance with the recommendations in the Guide for the Care and Use of Laboratory Animals of the National Institutes of Health. The protocol was approved by the School of Veterinary Medicine Animal Care and Use Committee at the University of Wisconsin (Permit Number: V910). Bucal swabs were obtained from each deer and the *PRNP* genes amplified and sequenced as described previously [4].

The deer were dosed daily, for five consecutive days, with 20 ml 10% (w/v) pooled brain homogenate. The pooled brain homogenate was prepared from obex brain samples obtained from two CWD-positive Wisconsin hunter-harvested deer. These deer were both wt/wt with respect to *PRNP* genotype and were histologically scored as stage 4 positive in the obex [4]. The inoculum was prepared in phosphate-buffered saline and was mixed with two cups deer pellet feed and fed to the deer. Additional feed was withheld from the deer for the five days of oral infection to ensure the complete consumption of inoculum.

Brains from each animal were homogenized (20% w/v) in cold PBS (DNase I 250 µg/ml) in a blender and then passed through different size needles. Aliquots of brain homogenate from each genotype were digested with proteinase K (50 µg/ml) for 30 minutes at 37°C, reactions were stopped by boiling in SDS sample buffer at 95°C for 10 minutes. The samples were resolved by western blot, using 12% NuPAGE Bis-tris gels (Invitrogen, CA) and PVDF membrane (Millipore). Blocking was performed in 5% milk in 0.1% TBS-T for 1 h at room temperature. Incubation with primary antibody, 8G8 1∶5000 (Cayman Chemical), was performed overnight at 4°C and HRP secondary antibody was used at 1∶10,000. Images were captured in a Typhoon system after ECL substrate addition (Pierce).

Obex immunohistochemistry was performed as described [4]. Briefly, samples were fixed in 10% neutral buffered formalin, dehydrated and embedded in paraffin. Tissue sections (5 µm thick) were cut and placed on positively charged slides. Slides were deparaffinized and antigen retrieval was performed by hydrated autoclaving in retrieval buffer. The tissue sections were exposed to anti-PrP mAb 6H4 (Prionics, Switzerland). Primary antibody was detected using a biotinylated secondary anti-mouse antibody, followed by horseradish peroxidase–streptavidin conjugate, chromagen substrate and hematoxylin counterstain.

### Statistical Analysis

An analysis of variance (ANOVA) was performed with survival time as outcome and membership in one of three groups (two copies of the wt *PRNP* allele; one copy each of the wt and G96S alleles; and one Q95H and one other allele) as a factor. The two subjects lost to follow-up because of intercurrent disease were omitted from analysis. This simplifies but does not bias the results because the premature deaths were judged unrelated to *PRNP* allele status and they occurred earlier than any deaths due to disease.

## References

[1] Tranulis MA (2002). Influence of the prion protein gene, PRNP, on scrapie susceptibility in sheep.. Apmis.

[2] Carlson GA, DeArmond SJ, Torchia M, Westaway D, Prusiner SB (1994). Genetics of prion diseases and prion diversity in mice.. Philos Trans R Soc Lond B Biol Sci.

[3] Prusiner SB, DeArmond SJ (1991). Molecular biology and pathology of scrapie and the prion diseases of humans.. Brain Pathol.

[4] Johnson C, Johnson J, Vanderloo JP, Keane D, Aiken JM (2006). Prion protein polymorphisms in white-tailed deer influence susceptibility to chronic wasting disease.. J Gen Virol.

[5] O'Rourke KI, Spraker TR, Hamburg LK, Besser TE, Brayton KA (2004). Polymorphisms in the prion precursor functional gene but not the pseudogene are associated with susceptibility to chronic wasting disease in white-tailed deer.. J Gen Virol.

[6] O'Rourke KI, Besser TE, Miller MW, Cline TF, Spraker TR (1999). PrP genotypes of captive and free-ranging Rocky Mountain elk (Cervus elaphus nelsoni) with chronic wasting disease.. J Gen Virol.

[7] Williams ES (2005). Chronic wasting disease.. Vet Pathol.

[8] Fox KA, Jewell JE, Williams ES, Miller MW (2006). Patterns of PrPCWD accumulation during the course of chronic wasting disease infection in orally inoculated mule deer (Odocoileus hemionus).. J Gen Virol.

[9] Sander P, Hamann H, Drogemuller C, Kashkevich K, Schiebel K (2005). Bovine prion protein gene (PRNP) promoter polymorphisms modulate PRNP expression and may be responsible for differences in bovine spongiform encephalopathy susceptibility.. J Biol Chem.

[10] O'Neill GT, Cairns D, Toovey L, Goldmann W, Hunter N (2005). New ovine PrP gene haplotypes as a result of single nucleotide polymorphisms in the PrP gene promoter.. J Anim Breed Genet.

[11] Grear DA, Samuel MD, Langenberg JA, Keane D (2006). Demographic patterns and harvest vulnerability of chronic wasting disease infected white-tailed deer in Wisconsin.. Journal of Wildlife Management.

[12] Mathiason CK, Powers JG, Dahmes SJ, Osborn DA, Miller KV (2006). Infectious prions in the saliva and blood of deer with chronic wasting disease.. Science.

[13] Haley NJ, Seelig DM, Zabel MD, Telling GC, Hoover EA (2009). Detection of CWD prions in urine and saliva of deer by transgenic mouse bioassay.. Plos One.

[14] Haley NJ, Mathiason CK, Zabel MD, Telling GC, Hoover EA (2009). Detection of Sub-Clinical CWD Infection in Conventional Test-Negative Deer Long after Oral Exposure to Urine and Feces from CWD plus Deer.. Plos One.

[15] Angers RC, Kang HE, Napier D, Browning S, Seward T (2010). Prion strain mutation determined by prion protein conformational compatibility and primary structure.. Science.

[16] Wilson GA, Nakada SM, Bollinger TK, Pybus MJ, Merrill EH (2009). Polymorphisms at the PRNP gene influence susceptibility to chronic wasting disease in two species of deer (Odocoileus Spp.) in western Canada.. J Toxicol Environ Health A.

